# Microdome-Tunable Graphene/Carbon Nanotubes Pressure Sensors Based on Polystyrene Array for Wearable Electronics

**DOI:** 10.3390/ma14237385

**Published:** 2021-12-02

**Authors:** Xingjie Su, Chunli Luo, Weiguo Yan, Junyi Jiao, Dongzhou Zhong

**Affiliations:** 1 School of Control and Mechanical Engineering, Tianjin Chengjian University, Tianjin 300384, China; abcsxj666@163.com (X.S.); junyi_0614@163.com (J.J.); 2 School of Science, Tianjin Chengjian University, Tianjin 300384, China; yanweiguo@tcu.edu.cn; 3 School of Information Engineering, Wuyi University, Jiangmen 529020, China

**Keywords:** resistive pressure sensors, self-assembly, polystyrene microspheres, tunable sensitivity

## Abstract

Resistive pressure sensors are appealing due to having several advantages, such as simple reading mechanisms, simple construction, and quick dynamic response. Achieving a constantly changeable microstructure of sensing materials is critical for the flexible pressure sensor and remains a difficulty. Herein, a flexible, tunable resistive pressure sensors is developed via simple, low-cost microsphere self-assembly and graphene/carbon nanotubes (CNTs) solution drop coating. The sensor uses polystyrene (PS) microspheres to construct an interlocked dome microstructure with graphene/CNTs as a conductive filler. The results indicate that the interlocked microdome-type pressure sensor has better sensitivity than the single microdome-type and single planar-type without surface microstructure. The pressure sensor’s sensitivity can be adjusted by varying the diameter of PS microspheres. In addition, the resistance of the sensor is also tunable by adjusting the number of graphene/CNT conductive coating layers. The developed flexible pressure sensor effectively detected human finger bending, demonstrating tremendous potential in human motion monitoring.

## 1. Introduction

Skin-inspired wearable devices hold tremendous potential in smart portable electronics’ next generation due to their intriguing uses in human body movement monitoring, physiological signal detecting, soft robotics, and human–machine interfaces [1,2,3,4,5,6]. Among these wearable electronics, flexible pressure sensors based on various sensing mechanisms play an important role in detecting external pressure, for example, piezoelectricity [7,8,9], resistivity [10,11,12,13], and capacitance [14,15,16]. Due to their simplicity of signal processing and wide application range, resistive flexible pressure sensors have been extensively developed among these pressure sensors. Although great advancements have been made in the preparation of high-sensitivity pressure-sensing e-skins, the tradeoff between sensitivity and manufacturing cost has been the core of the research. 

Resistive pressure sensors rely on two main aspects, which respond to various pressures. On the one hand, it depends on the resistivity of the sensing material. On the other hand, the microstructure of resistive pressure sensors is critical in improving the sensitivity [3,17]. Among sensing materials, graphene/CNTs exhibit some special advantages, including high electrical conductivity, inherent and structural flexibility, chemical and thermal stability [18,19,20,21,22,23,24,25]. These materials demonstrate outstanding mechanical and electrical characteristics, which make them viable candidates for wearing strain/pressure sensors. For example, Ho et al., using graphene, prepared a transparent and stretchable electronic skin sensor array, integrated the temperature, humidity, and pressure sensors via layer-by-layer superposition [18]. Dahiya Ravinder et al. reported a study on self-produced, flexible and transparent graphene tactile skin. A flexible capacitive touch sensor based on graphene was fabricated [19]. Professor D.H. Kim, using single-walled CNTs, fabricated flexible and wearable capacitors, field-effect transistors, and gate logic devices or gate logic devices [25].

Introducing microstructure into the design of resistive pressure sensors is an important factor for improving sensitivity and decreasing stress concentration. Recently, several regular micro/nanostructures, including nanowire [26,27], pyramid [28,29], hemisphere [30,31], and microdome [32,33,34,35], were used to improve the sensitivity of resistive pressure sensors. For example, graphene films based on pyramidal microstructure arrays give the tactile sensors ultra-high sensitivity (−5.5 kPa^−1^) in a low-pressure range (<100 Pa) [28]; the sensitivity of the pressure sensor prepared using UV-patterned silver nanowire/polydimethylsiloxane (AgNW/PDMS) composite was 3.179 kPa^−1^ (<2 kPa) [36]. The flexible pressure sensor based on interlocking microdome pattern PDMS showed high sensitivity (−15 kPa^−1^, <100 Pa) at low pressure [37]. Table 1 lists the sensitivity and sensing mechanisms by previously reported sensors with the microstructure. However, the metal film covering the microstructure surface is very easy to break during the bending motion of the sensor. Therefore, the graphene and CNT network, as a sensing layer, can overcome some shortcomings in fabricating resistive pressure sensors.

Herein, we provide a unique and low-cost approach based on PS microsphere self-assembly and conductive solution drop coating to fabricate resistive pressure sensors. Graphene/CNT film as a conductive layer improves the bending resistance of a pressure sensor. PS microspheres increase the contact area of conductive film to improve the sensitivity. As a result, the pressure sensor’s sensitivity can be adjusted by modifying the feature size of the microstructure. Moreover, the resistance value of the flexible pressure sensor can be flexibly adjusted via changing the number of layers of the graphene/CNTs conductive coating. The flexible sensor was successfully used to detect finger bending motion signals, showing its great application potential in wearable health monitoring system.

## 2. Experimental Section

### 2.1. Materials 

PDMS was purchased from Dow Corning (Sylgard 184). The PS solution had diameters of 2 μm and 5 μm (Huge biotechnology Co., Ltd., Shanghai, China). Multi-walled CNTs (outer diameter 8–15 nm, inner diameter 3–5 nm, length 3–12 μm, specific surface area > 232 m^2^/g, resistivity 1412 μΩm, purity > 95 wt%) and graphene (purity > 90 wt%, thickness ~2 nm, lamellar diameter < 10 μm) were purchased from Tanfeng Tech Co. Ltd., Jiangsu, China.

### 2.2. Fabrication of the PDMS Film

The liquid PDMS monomer and curing agent were mixed with the weight ratio of 10:1, then mechanically stirred for 10 min with a glass rod, and the mixture was left standing for 30 min to remove bubbles. Then, it was heated for 20 min in an air-blast drying oven at 80 °C to obtain an elastomer layer with a thickness of 2 mm. Then, the PDMS film was treated with oxygen plasma for 1 min to form a hydrophilic surface.

### 2.3. Preparation of the Monolayer PS Spheres Array 

**Figure 1:**
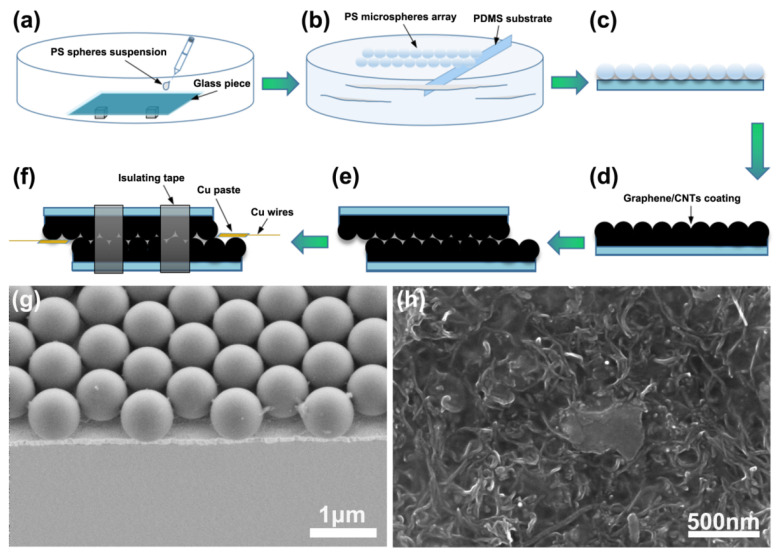
Sensor design and characterization. (**a**) Self-assembly of PS microspheres. (**b**) Assembling the microsphere on the PDMS substrate. (**c**) PDMS film coated with PS microsphere array. (**d**) Coating graphene/CNTs conductive solution on PS microsphere array. (**e**) Stack the two conductive sheets as shown in (**d**). (**f**) Flexible pressure sensor based on graphene/carbon nanotubes. (**g**) Scanning electron micrograph (SEM) of monolayer PS microsphere array. (**h**) SEM image of the surface of graphene/CNTs conductive coating.

First, we cleaned the glass with detergent to increase the hydrophilicity of the surface. Then, the monodisperse PS spherical suspension (10 wt% in ethanol) was ultrasonically treated at a frequency of 40 kHz for 10 min. Then, the deionized water was dropped onto the clean glass substrate in the vessel to form a water film covering the whole glass surface. Next, we dropped the PS sphere suspension into the water surface and it self-assembled into a monolayer PS sphere array with a large area and close arrangement (Figure 1a). Then, clean water was injected into the vessel to float the PS array on the water surface in the vessel. Next, the PDMS substrate, which was bombarded with oxygen plasma, was held with tweezers and placed underneath the PS film floating on the water surface. Finally, the PDMS substrate slowly lifted up underneath and the monolayer PS sphere array was transferred to the top of the PDMS sheet (Figure 1b,c).

### 2.4. Preparation of Graphene/CNTs Conductive Coating 

Before pouring onto the monolayer PS spheres array, a weight ratio of 2:1 (graphene to carbon nanotube) was well mixed (Figure 1d). To guarantee that the coating’s thickness was consistent, it was necessary to dip into a small amount of the graphene/CNTs solution with a thin plastic rod and apply it evenly on the PS microsphere array. Then, the sample was put into a 60 °C oven (DL-101, Zhonghuan Experimental Electric Furnace Co., Ltd., Tianjin, China) for 10 min.

### 2.5. Assembly of the Sensor 

The top layer of the graphene/CNTs was attached with copper paste and copper wire to facilitate the electrical performance measurement of the pressure sensor. The electrode was placed on one side of the graphene/CNTs conductive coating, the upper and lower plates were interlocked, and we stuck on the insulating tape to obtain a flexible pressure sensor (Figure 1e,f).

### 2.6. Characterizations of Graphene/CNTs Pressure Sensor 

The morphologies and microstructures of the conductive coating were comprehensively studied using a field emission scanning electron microscope (FESEM) (Ultra Plus, Zeiss, Oberkochen, Germany) and Raman spectroscopy (532 nm laser source, XploRA, HORIBA Jobin Yvon, Paris, France). A UNI-T UT804 multimeter was used to test the resistance. A semiconductor parameter analyzer was used to assess the sensors’ current-voltage (I–V) properties (4200A-SCS, Keithley, MO, USA).

### 2.7. Feasibility Analysis 

First, regarding the preparation of microstructure arrays, we obtained monolayer PS microsphere arrays by simple self-assembly techniques, but traditionally, microstructures are obtained by Si micro-structured mold flip. The disadvantage is that the manufacturing of a silicon microstructure mold is relatively more difficult, which is highly dependent on the equipment and complicated manufacturing processes, such as exposure, soft baking, development, hard baking, photoresist coating, etching and stripping photoresist. Then, considering the manufacturing cost, when preparing the PS microsphere array, the main materials we need are a simple water tank, an ordinary glass sheet, 60–80 μL of monodispersed PS suspensions, and 60–160 μL of anhydrous ethanol solution, which cost very little. In contrast, Si micro-structured arrays rely on a Si mold that is very costly to prepare; a piece of Si micro-structured mold with a size of 1.5 cm × 1.5 cm, for example, costs RMB 3000 to manufacture. Most critically, regarding the flexibility of microstructure size regulation, the morphology of the microstructure layer of our prepared sensor can be tuned through tailoring monodispersed PS microspheres’ diameter. As a result, the sensitivity of the pressure sensor may be altered by modifying the feature size of the microstructure. However, a Si micro-structured mold with microstructured surfaces may be utilized directly to recreate the microstructured patterns. Because of their inherent properties, the geometric parameters of microstructures are difficult to modify. A comparison regarding the manufacturing complexity, cost, PS microsphere size control and flexibility is shown in Table 2.

## 3. Results and Discussion

### 3.1. The Performance of the Graphene/CNTs Pressure Sensor 

**Figure 2:**
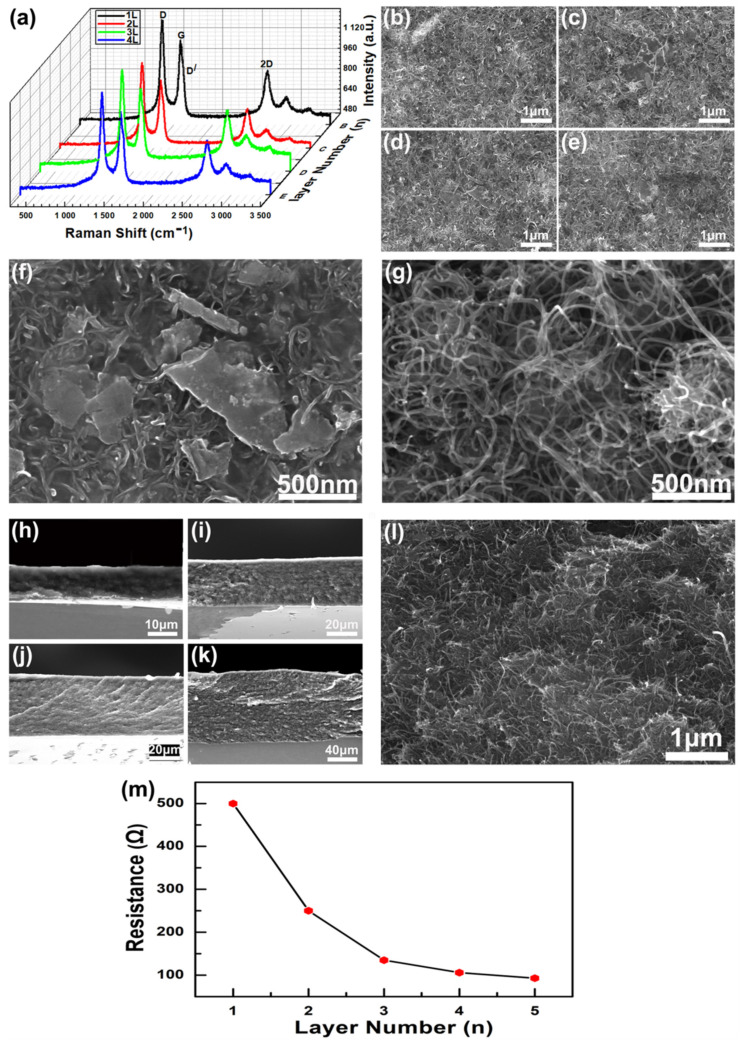
Characterization of graphene/CNTs conductive coating. (**a**) Raman spectra of graphene/carbon nanotube conductive coatings with different layers, respectively. (**b**–**e**) SEM image of 1~4-layer graphene/CNTs conductive coating surface. (**f**) SEM image of sheet-like multilayer graphene on conductive coating. (**g**) SEM image of multi-walled carbon nanotubes on conductive coating. (**h**–**k**) SEM side view of 1–4 layer graphene/CNTs conductive coating. (**l**) High magnification SEM side view of the conductive coating. (**m**) Relationship between the number of conductive coating layers and the resistance value of conductive layer.

A monolayer PS microsphere array is prepared by microsphere self-assembly technology, and then transferred to a PDMS sheet, thereby obtaining a flexible substrate with a uniform dome-shaped microstructure; a single-layer PS microsphere array is shown in Figure 1g. The graphene/CNTs coating has excellent conductivity; Figure 1h shows the SEM view image of the coating. The Raman spectrum of graphene/CNTs conductive coating shows the characteristic spectrum with three main peaks centered at 1334, 1585, and 2691 cm^−1^, which can be attributed to the D, G, and 2D bands, respectively (Figure 2a). The G band is due to the in-plane E2g mode, which arises from the stretching of the C–C bond, while D and D′bands can be attributed to the defects at the graphite edges.

**Figure 3:**
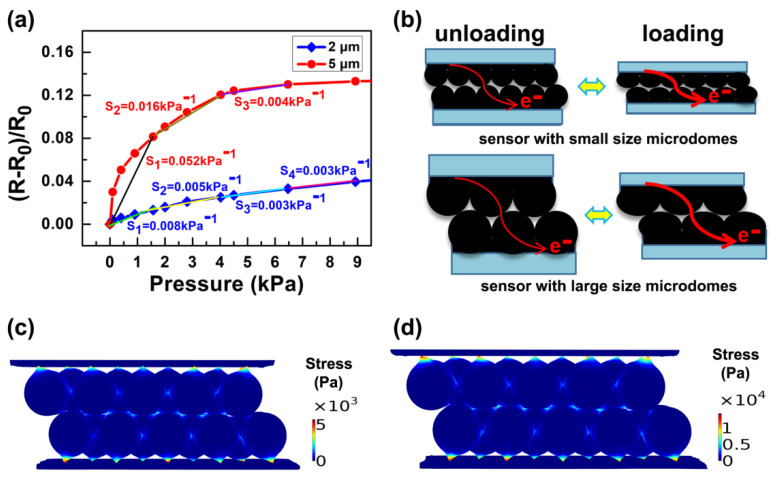
(**a**) Sensitivity of different pressure sensors based on 2 µm and 5 µm sized PS sphere. (**b**) Schematic illustration of the alterations that occur when the equal external pressure is applied to the pressure sensors based on 2 µm and 5 µm sized PS spheres. (**c**,**d**) Finite element modeling of the stress distribution and deformation of the sensors based on 2 µm and 5 µm diameter PS microspheres at 1 kPa applied pressure.

**Figure 4:**
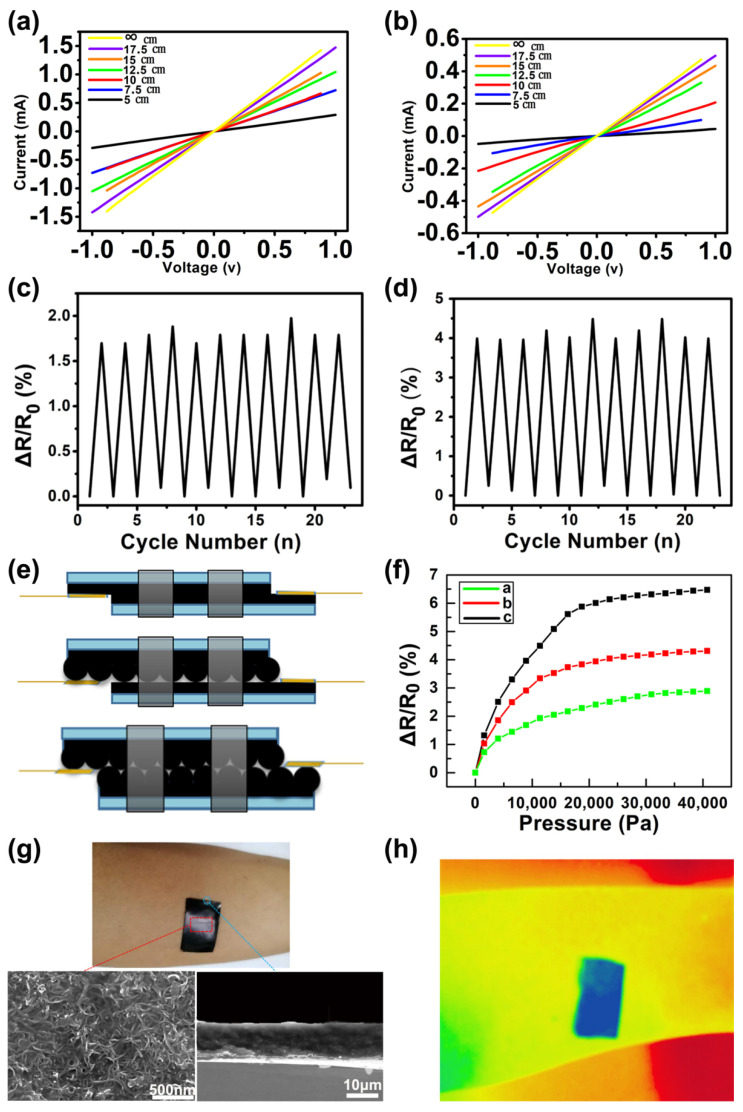
Performance test of graphene/CNTs pressure sensor. (**a**) The current-voltage (I–V) curve of a single planar pressure sensor without surface microstructure under different bending radii. (**b**) The I–V curve of the interlocking microdome pressure sensor under different bending radii. (**c**) The resistance change rate of a single planar pressure sensor without surface microstructure during cyclic bending. (**d**) The resistance change rate of the interlocking microdome pressure sensor during cyclic bending. (**e**) Pressure sensors of a single planar type without surface microstructure, a single microdome type, and an interlocking microdome type are all available. (**f**) The resistance change rate of pressure sensors with three different structures under pressure. (**g**) Test of the adhesion of the PDMS film with graphene/CNTs conductive coating to human skin. (**h**) Test of the infrared absorption effect of PDMS film with graphene/CNTs conductive coating.

**Figure 5:**
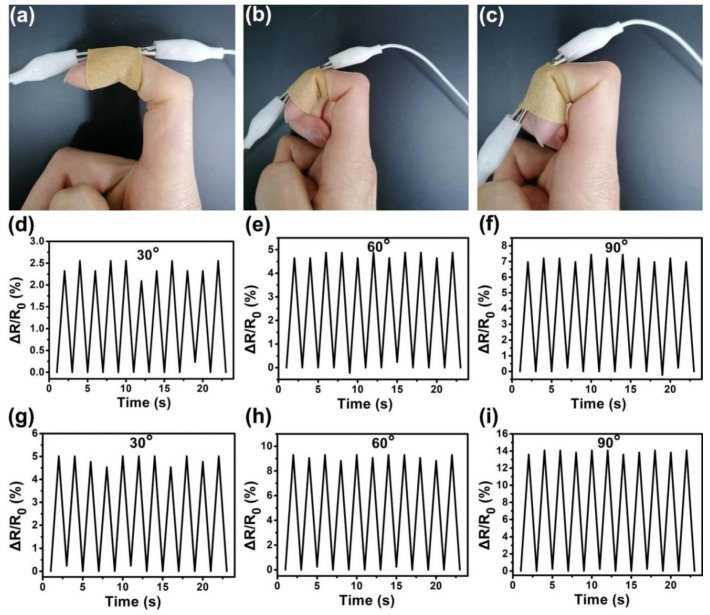
Response of pressure sensor to real-time human motions. (**a**–**c**) Pressure sensor fixed on the finger with a band-aid to monitor the bending motion of the joint at different angles. (**d**–**f**) The resistance change rate of a single planar pressure sensor with no surface microstructure when the finger performs 30, 60, and 90 degree cyclic bending motions. (**g**–**i**) The resistance change rate of the interlocking micro-dome pressure sensor when the finger performs 30, 60, and 90 degree cyclic bending motions.

**Figure 6:**
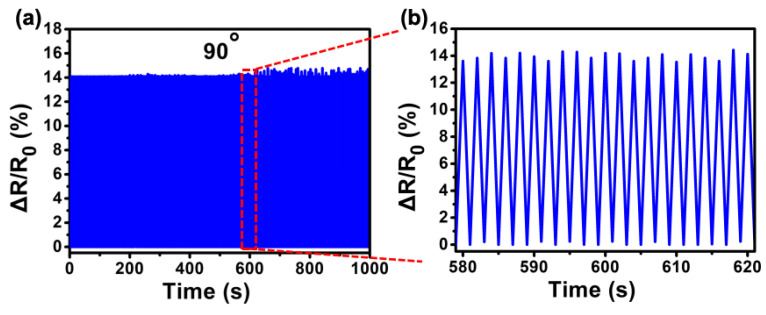
(**a**) The sensor is tested for 90° bending for 480 cycles. (**b**) The 21 cyclic tests extracted from the red region in (**a**).

The morphology and microstructure of the surface of 1–4 layers of graphene/CNTs conductive coating were characterized by the SEM top view image (Figure 2b–e). It can be seen that the surface morphology and microstructure of different layers of conductive coatings are different. Specifically, a conductive coating with a larger number of layers has a bulk conductor formed by stacking more graphene, as shown in Figure 2f. In addition, the multi-walled CNTs contained in the conductive coating are intertwined and woven together, as shown in Figure 2g. The greater the number of conductive coating layers, the more CNTs are contained, and the tighter the conductive mesh is interwoven. Figure 2h–k shows the SEM side views of 1–4 layers of graphene/CNTs conductive coatings respectively, where 2l is a side view with a larger magnification. Obviously, the coatings between adjacent layers are tightly bonded. With the increase in the number of graphene/CNT conductive coating layers, the conductivity is enhanced. In particular, in the process of increasing the number of layers from integer 1 to integer 3, the resistance is dropped significantly. This is because the two conductive materials, graphene and CNTs, combine more densely. Furthermore, it should be noted here that the sensitivity of the sensor is mainly controlled by the size of the PS microspheres. The increase in the number of conductive layers will slightly reduce the sensitivity of the sensor and slightly improve the mechanical strength, but it has little effect. Although increasing the number of conductive layers will enhance the conductivity of conductive layers, that is, the resistance will decrease, the resistance is not directly related to the sensitivity or mechanical strength. As shown in Figure 2m, it is the relationship between the number of conductive coating layers and the sensor resistance. Although the resistance value can be changed by adjusting the number of conductive layers, considering the cost and performance comprehensively, if there is no extremely high requirement for the conductivity of the conductive layers, the performance of the sensor made of a single conductive layer is good enough. Therefore, the pressure sensors in Figure 3, Figure 4, Figure 5 and Figure 6 are all made of a single conductive layer.

The pressure-sensing abilities of the manufactured pressure sensors based on 5 µm and 2 µm diameter PS spheres are investigated by measuring relative resistance changes. The pressure sensitivity (S) may be calculated using the formula $S=\;\delta (\Delta R/R_{0})/\delta P$, where P signifies the applied pressure, and R and R_0_ signify the resistance change with load pressure and beginning resistance without pressure or load, respectively. The relative differences in resistance of the pressure sensors based on 5 µm and 2 µm diameter PS sphere are shown in Figure 3a. When the sensor is subjected to the same pressure, the microstructure film with the bigger characteristic size will experience more significant deformation and a large relative change in resistance, as shown in Figure 3b. To explore the dimensional influence of dome-shaped structures on sensor performance systematically, the sensitivity within the low-pressure region may be approximated as:

$$S_{sen}=\frac{H}{S_{0}E}\times 2\Pi r=\frac{\Pi }{4S_{0}E}\times \left(D^{2}+4H^{2}\right)$$

where $S_{0}$ is the initial contact area between the microdomes, *D* is the dome’s diameter, *H* is the dome’s height, and *E* is the elastic modulus of PS. The height and diameter of the dome have a positive effect on the sensitivity in the low pressure range, which is clearly presented in the formula. Figure 3c,d depicts the stress distribution of the finite element simulated interlocked microdome sensor at 1 kPa applied pressure. Here, the monolayer microstructured films with microsphere diameters of 2 µm and 5 µm are assembled into interlocking dome pressure sensors in turn. The stress distribution graphic illustrates that as the load rises, the contact area *S* between interlocking microdomes grows and the dome height *H* decreases. Under the load applied state, the local stresses are focused on the contact faces between the interlocked microdomes. In addition, compared with the sensor with a microsphere diameter of 2 µm, the stress distribution range of the sensor with a microsphere diameter of 5 µm is wider. 

In order to more clearly discuss the role of the PS microsphere size in the work, Table 3 records the sensitivity values of pressure sensors based on PS microspheres of 5 μm and 2 μm in different pressure ranges in detail. At low pressure (<1600 Pa), the sensitivity of the sensor based on 2 µm diameter PS microspheres is 0.00825 kPa^−1^, while the sensitivity of the sensor based on 5 µm PS microspheres is as high as 0.05194 kPa^−1^, the latter being more than 6 times more sensitive than the former. In particular, when the pressure is less than 100 Pa, the sensitivity of them is 0.04 kPa^−^^1^ and 0.3 kPa^−^^1^ respectively, the sensitivity is higher, and the disparity between them is larger. This is because the pressure sensor based on large PS microspheres can cause more severe deformation of the conductive film under the same pressure because of the larger size of the microspheres, while the sensor based on small size PS microspheres cannot cause obvious deformation and larger relative resistance change due to the size of the microspheres being much smaller than the thickness of the conductive film. In the medium pressure range (1600–4000 Pa), the sensitivity of the sensor based on small microspheres is 0.00495 kPa^−1^, while that based on large microspheres is 0.01624 kPa^−1^. Compared with the low pressure state, the sensitivity of PS microspheres is decreased to a greater extent, which is because the deformation of the PS microspheres is limited in a certain range and nonlinear, and the deformation of the PS microspheres is larger under the initial pressure. After increasing the pressure, the deformation increment gradually decreases, due to its own rigidity. On the other hand, because PS microspheres are covered on the flexible substrate of PDMS, the external pressure exerted on the sensor surface will be conducted down to the substrate through the PS microspheres. When the pressure is low, the substrate will sag down, which will cause greater bending deformation of the conductive film. However, as the pressure continues to increase, the upward reaction force of the substrate to the microspheres will also increase, and the deformation increment of the substrate will also decrease significantly. In the high pressure range (4000~6500 Pa), the sensitivities of both are 0.00317 kPa^−1^ and 0.00389 kPa^−1^, respectively. With the increase in external pressure, the sensitivity of the sensor based on large-size microspheres decreases more rapidly, as, at this time, the sensitivity of both sensors is almost equal, and the sensor based on large-size microspheres reaches saturation pressure. The reason for the difference in sensitivity of pressure sensors based on different PS sphere sizes is related to the change in surface area of the conductive film. The microstructure conductive film with smaller feature size covers a large number of microspheres in the same area, and the microstructure array composed of microspheres with a smaller size is arranged more tightly, has a larger surface area, and has stronger resistance to pressure. When the same pressure is applied, the deformation, contact area and resistance change of the sensor are smaller. At a higher pressure (6500~8900 Pa), because the sensor based on large-size microspheres has reached saturation pressure, it cannot respond to external pressure efficiently. However, the sensitivity of the sensor based on small-sized microspheres decreases relatively slowly with the increase in pressure, so the saturation pressure is higher, and the sensitivity is 0.00275 kPa^−1^ at this time. In summary, it is clear that the feature size of PS microspheres has a strong modulating effect on the sensitivity and pressure detection range of the sensor as well as the corresponding regulation mechanism.

Figure 4a,b shows the pressure sensor’s current–voltage (I–V) curves at the various radius of curvature. As the voltage is swept from −1 V to 1 V, the applied pressure remains constant. The slopes of the I–V curves reduce as the degree of bending increases, showing that resistance increases as curvature increases. The linearity of the I–V curves implies that Ohm’s contact properties dictate the device’s behavior. Among them, Figure 4a is the I–V curve of the pressure sensor without the PS microsphere array, and Figure 4b is the I–V curve of the PS microsphere array pressure sensor with a diameter of 2 μm. The resistance of the former increases by 11 times during the process from natural extension to bending to a radius of curvature of 5 cm. In the latter, under the same conditions, the resistance is increased by 5 times. Obviously, the interlocked microstructured sensor is more sensitive than the planar surface sensor. The curve of the resistance change rate of the pressure sensor without the PS microsphere array during the bending process is shown in Figure 4c, and Figure 4d is the resistance change rate curve of the pressure sensor with a 2 μm diameter PS microsphere array during the bending process. Three different pressure sensors were prepared, namely, single plane type, single microdome type and interlocking microdome type based on 2 μm diameter microspheres (Figure 4e), in order to study the influence of the surface micro-structure on sensor sensitivity. Figure 4f shows a comparison of their electrical resistance changes when subjected to external pressure, corresponding to the three curves: a, b, and c. Although all of them display an increase in resistance as pressure rises, the interlocking microdome sensors’ responses differ significantly from those of the planar sensor. In reaction to pressure, we can notice a considerable reaction for the microstructured sensors. When the pressure is applied to 18,000 Pa, the resistance change rate of the interlocked microdome sensor is 5.87%, which is significantly higher than the resistance change rate of the non-structured sensor 2.29% and the single-microstructured sensor 3.83%. However, when the applied pressure is in the range of 18,000~40,000 Pa, as the pressure increases, the sensor resistance increases relatively slowly and tends to be stable. The PDMS flexible substrate of the pressure sensor has good adhesion to human skin (Figure 4g). In addition, as shown in Figure 4h, the graphene/CNTs hybrid coating of the pressure sensor can effectively absorb infrared rays. Therefore, the flexible pressure sensor possesses extensive use outlooks in wearable medical monitoring devices, electronic skin, artificial intelligence, and soft robotics [23].

### 3.2. The Specific Application of the Graphene/CNTs Pressure Sensor 

The graphene/CNTs pressure sensor was fixed on the finger through a band-aid to monitor the bending motion of the joint at different angles (Figure 5a–c). At the same time, real-time resistance changes were recorded (Figure 5d–i). The resistance change rate of the pressure sensor without the PS microsphere array in the cyclic bending of the finger at different angles is shown in Figure 5d–f. Figure 5g–i corresponds to the resistance change rate of the pressure sensor with a 2 μm diameter PS microsphere array. It was found that the resistances of the sensor showed corresponding increases or decreases with the deformation of the finger. Furthermore, under the same conditions, the resistance change rate of the interlocking microstructure sensor was almost twice that of flat surface sensor.

There is a significant difference in sensitivity between them, providing more contact area under the same applied pressure, which is the reason why the interlocking microstructure sensor is far more sensitive than the sensor with flat surface. As a consequence, as compared to a pressure sensor composed of an unstructured substrate, the structured sensor’s sensitivity to outside pressure may be effectively boosted. The decreased contact resistance between the two interlocked conductive films caused by the increased contact area under outside load is the main reason for this result. The amazing sensing performance of our sensor is due to the changing in the contact zone, which is generated by the deformation of the microstructure.

To investigate the long-term stability, a pressure sensor based on a 2 μm diameter PS sphere was fixed on the finger and bent at 90°/released 480 times. As shown in Figure 6a, after 480 cycles, the change in relative resistance showed almost no change, and only after the 290th cycle, the change rate of resistance increased slightly during bending. Figure 6b shows 21 random cycle tests extracted from the red region in Figure 6a; the curves of each bending–releasing cycle are almost the same, and the high reproducibility and durability of the microstructure sensor are proved by the very similar amplitude and waveform. We ascribe this exceptional endurance to the PS microspheres’ and PDMS substrate’s strong elasticity, which can resist numerous mechanical deformation cycles.

## 4. Conclusions

In summary, a novel graphene/CNTs resistive pressure sensor based on interlocking microdome structure was successfully fabricated and showed to be significantly improved in adjusting sensitivity. Graphene/CNTs are used as the conductive layer to enhance the bending resistance of the sensor, and the PS microsphere array changes the contact area of the conductive film to adjust the sensitivity. By introducing PS microspheres with a larger feature size, the sensitivity of the sensor was significantly improved. The resistive pressure sensor was successfully used for real-time monitoring of finger bending motion. In addition, 480 cycles of the bending test were carried out on the pressure sensor fixed on the finger; the consistency of the curve of relative resistance change rate showed that the sensor has high stability and good durability. Therefore, this work provides a novel strategy for manufacturing flexible pressure sensors with high performance and low cost through the use of carbon nanomaterials and microstructure construction.

## Figures and Tables

**Table 1:** Comparison of the sensitivity based on this work and previous reports.

Reference	Sensitivity (kPa^−1^)	Pressure Range (kPa)	Sensing Mechanism
[35]	0.533	0–2	Resistance
[36]	0.438	0–2	Resistance
[26]	0.034	0.1< or >10	Capacitive
[23]	0.0115	0–30	Capacitive
[37]	0.23 × 10^−3^	0–3000	Resistance
This work	0.02	0–6.5	Resistance

**Table 2:** Comparison of manufacturing complexity, cost, and flexibility in size control of PS microspheres.

	Microarray (1.5 cm × 1.5 cm)	
	PS Microspheres	Silicon Template
Manufacturing complexity	simple (self-assembly technology)	complex (photolithography)
Cost	low (RMB 2)	high (RMB 3000)
Size control flexibility of microstructure	easy to adjust geometric parameters	difficult to adjust geometric parameters

**Table 3:** Experimental sensitivity values of the pressure sensor.

	Corresponding Pressure (Pa)			
Size of the microdomes (kPa^−1^)	0–1600	1600–4000	4000–6500	6500–8900
2 μm	0.00825	0.00495	0.00317	0.00275
5 μm	0.05194	0.01624	0.00389	0.0012

## Data Availability

The data presented in this study are available on request from the corresponding author.
